# Downregulation of a putative plastid PDC E1α subunit impairs photosynthetic activity and triacylglycerol accumulation in nitrogen-starved photoautotrophic *Chlamydomonas reinhardtii*

**DOI:** 10.1093/jxb/eru374

**Published:** 2014-09-10

**Authors:** Nastassia Shtaida, Inna Khozin-Goldberg, Alexei Solovchenko, Konstantin Chekanov, Shoshana Didi-Cohen, Stefan Leu, Zvi Cohen, Sammy Boussiba

**Affiliations:** ^1^Microalgal Biotechnology Laboratory, French Associates Institute for Agriculture and Biotechnology of Drylands, J. Blaustein Institutes for Desert Research, Ben-Gurion University of the Negev, Sede Boqer Campus 84990, Israel; ^2^Department of Bioengineering, Faculty of Biology, Moscow State University, 119234, GSP-1 Moscow, Russia

**Keywords:** Acetyl-CoA, chlorophyll fluorescence transients, fatty acid synthesis, lipid, microalgae, photosystem II, pyruvate dehydrogenase.

## Abstract

The chloroplast pyruvate dehydrogenase complex (cpPDC) catalyses the oxidative decarboxylation of pyruvate forming acetyl-CoA, an immediate primer for the initial reactions of *de novo* fatty acid (FA) synthesis. Little is known about the source of acetyl-CoA in the chloroplasts of photosynthetic microalgae, which are capable of producing high amounts of the storage lipid triacylglycerol (TAG) under conditions of nutrient stresses. We generated *Chlamydomonas reinhardtii* CC-1618 mutants with decreased expression of the *PDC2_E1α* gene, encoding the putative chloroplast pyruvate dehydrogenase subunit E1*α*, using artificial microRNA. A comparative study on the effects of *PDC2_E1α* silencing on FAs and TAG production in *C. reinhardtii*, grown photoautotrophically and mixotrophically, with and without a nitrogen source in the nutrient medium, was carried out. Reduced expression of *PDC2 _E1α* led to a severely hampered photoautotrophic growth phenotype with drastic impairment in TAG accumulation under nitrogen deprivation. In the presence of acetate, downregulation of *PDC2_E1α* exerted little to no effect on TAG production and photosynthetic activity. In contrast, under photoautotrophic conditions, especially in the absence of a nitrogen source, a dramatic decline in photosynthetic oxygen evolution and photosystem II quantum yield against a background of the apparent over-reduction of the photosynthetic electron chain was recorded. Our results suggest an essential role of cpPDC in the supply of carbon precursors for *de novo* FA synthesis in microalgae under conditions of photoautotrophy. A shortage of this supply is detrimental to the nitrogen-starvation-induced synthesis of storage TAG, an important carbon and energy sink in stressed *Chlamydomonas* cells, thereby impairing the acclimation ability of the microalga.

## Summary

Impaired carbon precursor supply through cpPDC is detrimental for TAG biosynthesis in *Chlamydomonas reinhardtii* under conditions of photoautotrophy and nitrogen starvation.

## Introduction

Elucidation of the storage lipid metabolism in oleaginous photosynthetic microalgae is attracting major attention from the research community, given the potential capacity of these organisms to produce precursors for the biodiesel industry and the recently revealed peculiar traits of microalgal lipid metabolism (Liu and Benning, 2013; Goodenough *et al.*, 2014; Li *et al.*, 2014). This focus on microalgal lipid metabolism necessitates a deep understanding of the key regulatory factors and the major pathways implementing carbon allocation to fatty acid (FA) synthesis in the chloroplast, and subsequent processing towards the assembly of reserve lipids, mainly triacylglycerols (TAG). Enzymes involved in the direct conversion of pyruvate, originating from glycolysis, and some coupled reactions utilizing pyruvate and phosphoenolpyruvate (PEP)—the so-called pyruvate hub enzymes—have been proposed as promising targets for metabolic engineering in microalgae to shuffle the carbon flux towards FA and TAG biosynthesis (Smith *et al.*, 2012; Jinkerson *et al.*, 2013). In higher plants (Chapman *et al.*, 2013), as well as in microalgae (Klein, 1986; Smith *et al.*, 2012), both cytosolic and plastidic glycolytic pathways are incomplete, and hence they complement each other and depend on metabolite trafficking across the plastid membrane (Facchinelli and Weber, 2011). Notably, the green microalga *Chlamydomonas reinhardtii* lacks the chloroplast pyruvate kinase, generating pyruvate from PEP (Klein, 1986; Terashima *et al.* 2011). The chloroplast pyruvate dehydrogenase complex (cpPDC) is known to convert pyruvate into acetyl-CoA to be used in FA synthesis.

The oxidative decarboxylation of pyruvate to acetyl-CoA occurs along with the commensurate production of NADH. PDC consists of three main components: E1 or pyruvate dehydrogenase (PDH; EC 1.2.4.1), E2 (dihydrolipoamide acetyltransferase, EC 2.3.1.12), and E3 (dihydrolipoamide dehydrogenase, EC 1.6.4.3). E1 is composed of alpha (E1α) and beta (E1β) subunits. The pyruvate decarboxylation reaction, catalysed by PDH, is considered to be a rate-limiting step in the reaction sequence mediating PDC activity (Reid *et al.*, 1977).

Acetyl-CoA is an important metabolite that participates in numerous anabolic reactions, as well as in protein acetylation, pinpointing the essential role played by PDC in overall cellular metabolism. Regarding the lipid biosynthesis pathways, much evidence suggests that plastid PDC plays a dominant role in providing acetyl-CoA for FA synthesis in the cells of oleaginous plant tissues and organs (Ke *et al.*, 2000; Lin *et al.*, 2003; Bourgis *et al.*, 2011). Direct evidence of the critical role of plastid PDC was obtained by disruption of the gene encoding the subunit E2 of the cpPDC, which led to an embryo-lethal phenotype in the developing seeds of *Arabidopsis* (Lin *et al.*, 2003). At the same time, alterations in the gene expression of plastid acetyl-CoA synthase (ACS), utilizing acetate to generate acetyl-CoA, did not lead to significant changes in lipid content in *Arabidopsis* developing seeds or leaves (Behal *et al.*, 2002; Lin and Oliver, 2008). Recent comparative transcriptome and metabolome studies of oil and date palms further indicated the plastid carbon metabolism and the acetyl-CoA supply via PDC for FA synthesis and, ultimately, for TAG biosynthesis as important determinants of oleogenity in higher plants (Bourgis *et al.*, 2011).

Regulation of storage lipid metabolism in photosynthetic unicellular algae is deemed to be distinct from that in the well-studied oil-storing tissues of land plants (Liu and Benning, 2013). The exposure to stress conditions, such as nitrogen starvation, arrests the cell cycle, hampers growth, and leads to a swift diversion of cellular metabolism from protein and nucleic acid synthesis to accumulation of reduced-carbon storage compounds, such as TAG, in the oleaginous species (Hu *et al.*, 2008; Solovchenko, 2012). The flexibility of algal lipid metabolism allows the rapid remobilization of TAG reserves upon nitrogen replenishment (Khozin-Goldberg *et al.*, 2005). Relatively little information is available on the source of acetyl-CoA in the chloroplasts of photoautotrophic oleaginous microalgae, capable of producing high amounts of the storage lipid TAG under conditions of nutrient stresses. Multiple enzymatic activities, not limited to the chloroplast, can potentially lead to acetyl-CoA production in microalgal cells other than PDC, such as ACS, acetate metabolism via acetate kinase and phosphotransacetylase, and the PDC bypass, involving pyruvate decarboxylase and aldehyde dehydrogenase, depending on the carbon and light regime (Li *et al.*, 2014; Goodenough *et al.*, 2014). [^14^C]acetate labelling studies, in various species, have demonstrated active acetate acquisition and incorporation into FA and complex lipids, implying ACS activity in microalgal cells. Moreover, the cellular content of TAG in *C. reinhardtii* was drastically augmented by increasing acetate concentration in the nutrient medium (Fan *et al.*, 2012). Recent studies have shed more light on the transcriptional regulation of TAG overproduction in a starchless mutant of *C. reinhardtii* with an acetate boost, including the increased expression of acetate transporters and two ACSs (Goodenough *et al.*, 2014).

There is no direct experimental evidence for the involvement of cpPDC in the supply of acetyl-CoA for *de novo* FA synthesis in microalgae under conditions of photoautotrophy, in general, and under conditions conducive to TAG accumulation, such as nitrogen starvation, in particular. However, much pertinent information on the abundance and patterns of gene expression of putative cpPDC-encoding genes is available from a rapidly growing number of genomic and transcriptomic studies on microalgae. Based on the transcriptional dynamics under nitrogen starvation, triggering FA and TAG biosynthesis, the transcriptional regulation of cpPDC genes is posited as an important mechanism regulating carbon flux from photoassimilates towards the biosynthesis of FA and TAG in photoautotrophic oleaginous microalgae (Rismani-Yazdi *et al.*, 2012; Jinkerson *et al.*, 2013; Li *et al.*, 2014).

Remarkably, the dual role of dihydrolipoamide acetyltransferase (DLA2), an E2 subunit of the cpPDC from *C. reinhardtii*, in the synthesis of both acetyl-CoA and D1 protein has recently been revealed (Bohne *et al.*, 2013). The reciprocal regulation of metabolic signals and gene expression relied on the carbon regime; in the presence of acetate, DLA2 could bind *psbA* mRNA favouring D1 protein synthesis and the *de novo* assembly of photosystem II (PSII).

Gaining further insight into the role of cpPDC in acetyl-CoA synthesis in photosynthetic microalgal cells is essential for a better understanding of the mechanisms regulating carbon flux and allocation for FAs and TAG biosynthesis in oleaginous microalgae. In the current study, *C. reinhardtii* recombinant lines with a decreased expression of *PDC2_E1α* (Cre02.g099850; http://www.phytozome.net), predicted to encode the subunit E1α of cpPDC, were generated using artificial microRNA (amiRNA) technology (Molnar *et al.*, 2009). Transformants were used in a comparative study of the role of cpPDC in FA and TAG production in the model green microalga *C. reinhardtii* under photoautotrophic and mixotrophic conditions in the presence and absence of nitrogen in the nutrient medium. Significantly impaired growth was observed in the selected lines grown with a CO_2_ supply as the only external carbon source. Under nitrogen starvation, the deleterious effect of decreased gene expression on TAG and biomass accumulation was profound under photoautotrophy. The analysis of oxygen evolution and electron transport on the acceptor side of PSII indicated an overall decline in photosynthetic activity during photoautotrophic growth, especially under nitrogen starvation, presumably due to a sink limitation resulting from TAG biosynthesis impairment. Based on these results, we assume that cpPDC is at least one control point in the supply of acetyl-CoA for *de novo* FA synthesis in the plastids of *C. reinhardtii* under photoautotrophic conditions, and this assumption may apply to other photoautotrophic microalgae as well. In addition, this mechanism seems to play an important role in acclimation to stresses such as nitrogen limitation by channelling of the excessively fixed carbon into the biosynthesis of storage TAG.

## Materials and methods

### Strains and experimental conditions

A *C. reinhardtii* cell-wall-deficient and arginine-requiring mutant strain (CC-1618 arg7 cw15 mt^–^) was obtained from the Chlamydomonas Resource Center (University of Minnesota, St Paul, MN, USA; http://chlamycollection.org/). Arginine was only supplied to the original strain before transformation; all media used for the engineered strains were arginine free. The following media were used in the experiments: nitrogen-replete and nitrogen-depleted (containing 0.5mM NH_4_Cl, 20 times less than the full medium) Tris/acetate/phosphate (TAP: 200mM Tris, 20mM NaAc) medium, and nitrogen-replete and nitrogen-depleted Sueoka’s high-salt minimal (HSM) medium (Harris, 1989).

*C. reinhardtii* strain CC-1618 cells were cultured in TAP with periodic dilutions with fresh medium to maintain logarithmic growth as estimated by daily measurements of the chlorophyll (Chl) culture content (Solovchenko *et al.*, 2013). At the onset of each experiment, cultures were diluted according to Chl content (initial Chl was 2 and 15 µg ml^–1^, cell number *~*1×10^6^ and 6×10^6^ cell ml^–1^ for the experiments in nitrogen-replete and nitrogen-depleted medium, respectively). Before transfer to nitrogen starvation, cells were washed twice with a nitrogen-depleted medium. All experiments were performed in 250ml Erlenmeyer flasks containing 100ml (TAP) or 70ml (HSM) liquid culture in a New Brunswick Innova 43 incubator shaker (Eppendorf AG, Hamburg, Germany) at 25 °C, with CO_2_ supplied at 300ml min^–1^ under continuous illumination (75 μmol photons m^–2^ s^–1^ photosynthetically active radiation). For photoautotrophic growth in HSM medium, the flasks were sealed with AirOtop enhanced seals (Thompson Instrument Company, Oceanside, CA, USA) to provide better gas exchange.

Cultures were sampled for analyses of growth parameters and lipids at the beginning of the experiment and after 2, 4, and 6 d for the experiments in complete medium, and after 2 d when experiments were conducted under nitrogen-starvation conditions. Total Chl concentration in the cultures was determined as described by Solovchenko *et al.* (2013). For dry weight (DW) measurements, two types of membrane filter were set together: glass fibre filters (Sartorius Stedim Biotech, Gottingen, Germany) with a pore size of 5–8 µm and cellulose-nitrate filters with a pore size of 5 µm (Schleicher & Schuell, Dassel, Germany), which were placed on top of the glass filters. *C. reinhardtii* culture (5ml) were filtered through the two dried and pre-weighed filters, and filters were dried for 120min at 120 °C or until a constant weight was achieved.

### Lipid extraction and FA analysis

Prior to lipid extraction, biomass samples that had been freeze dried and kept at –20 °C were treated for 10min with hot isopropanol at 80 °C to prevent lipase activity. Isopropanol was collected, and lipids were extracted from the cell pellets according to the method of Bligh and Dyer (1959) and combined with isopropanol extracts. Total lipid extract was separated into neutral (NLs) and polar (PL) lipids on Bond-Elute silica cartridges (Varian, Walnut Creek, CA, USA). TAG was isolated from NL using a solvent system of petroleum ether:diethyl ether:acetic acid (70:30:1, v/v); PL were separated into individual classes by two-dimensional thin-layer chromatography as previously described (Pal *et al.*, 2013).

A gas chromatography (GC) analysis of FA composition and content was performed on biomass samples, aliquots of lipid extracts, and individual lipid classes isolated by thin-layer chromatography according to Pal *et al.* (2013). Prior to the direct transmethylation, cell pellets were dried for 45min in a Savant SPD 111 SpeedVac concentrator (Thermo Electron) at room temperature. For transmethylation, the biomass and lipid samples were incubated in dry methanol containing 2% (v/v) H_2_SO_4_ at 80 °C for 1.5h under argon atmosphere and continuous stirring. Heptadecanoic acid (C17:0; Sigma-Aldrich) was added as an internal standard. FA methyl esters were analysed on a Trace GC ultra (Thermo, Milan, Italy) equipped with a SUPELCOWAX 10 (30 m×0.32mm, 0.25 μm) capillary GC column (Sigma-Aldrich), a flame ionization detector, and a programmed temperature vaporizing injector (Pal *et al.*, 2013).

### Vector construction for silencing PDC2_E1α and *Chlamydomonas* transformation

Gene silencing was performed using amiRNA as described by Molnar *et al.* (2009). The expression vector pChlamiRNA2, containing the amiRNA precursor under the hybrid HSP70A–RBCS2 promoter and *Arg7*, was obtained from the Chlamydomonas Resource Center. Using the web MicroRNA designer platform (WMD3; http://wmd3.weigelworld.org/cgi-bin/webapp.cgi), two complementary 90 nt oligonucleotides, containing 21 nt of the coding region of *C. reinhardtii PDC2_E1α* (Cre02.g099850, Phytozome), in forward and reverse orientations and separated by the spacer to form a loop structure, were designed (ami-PDC2_E1α-F and ami-PDC2_E1α-R; Supplementary Table S1 at *JXB* online). After annealing, the double-stranded oligonucleotides were ligated into the *SpeI*-digested pChlamiRNA2 vector using a Mighty Mix kit (Takara Bio, Otsu, Japan). The *C. reinhardtii* mutant strain CC-1618 arg7 cw15 mt^–^ was transformed with the resultant pChlami2-ami*pdh* vector using the glass-bead method (Kindle, 1990) and grown on TAP agar plates without arginine for selection. Individual colonies were detected after 1–1.5 weeks and transferred to fresh TAP plates. *C. reinhardtii* cells transformed with the original pChlamiRNA2 vector carrying *Arg7* were used in the experiments as an empty vector (EV) control.

### Selection of *pdh* transformants

Selection of transformants with decreased expression of *C. reinhardtii PDC2_E1α* (*pdh* lines) was based on their expected reduced ability to grow photoautotrophically. A total of 86 *pdh* colonies and 10 EV colonies were randomly selected and grown in 96-well culture plates in 200 µl of TAP medium. After a few days of growth, 50 µl of culture of each line was transferred to 24-well plates containing 950 µl of either TAP (mixotrophic conditions) or HSM (photoautotrophic conditions) medium. After 3 d of growth under continuous illumination (50 μmol photon m^–2^ s^–1^ photosynthetically active radiation), OD_750_ was measured using a microplate reader (BioTek, Winooski, VT, USA). Growth was evaluated by comparing the OD values of mutants in HSM and TAP relative to those of the EV lines. Lines exhibiting the lowest *pdh*OD_750 HSM/TAP_:EV OD_750 HSM/TAP_ ratio were selected as potential candidates carrying the silenced *PDC2_E1α.*

Selected lines were examined for the presence of the vector insert by PCR using primer pair pChlamiRNA2-F/pChlamiRNA2-R (Supplementary Table S2 at *JXB* online). DNA from different transformant lines was isolated using the fast CHELEX-based extraction procedure (Heddad *et al.*, 2006). A few PCR-positive clones were selected for RNA isolation and further analysis of target gene expression by real-time quantitative PCR (qPCR).

### RNA isolation, cDNA synthesis, and real-time qPCR

For RNA isolation, 3ml of logarithmically growing culture of the selected *pdh* and EV lines was centrifuged (13 000rpm, 5min, 4 °C), and the resultant cell pellet was processed with a SV Total RNA Isolation System kit (Promega, Madison, WI, USA).

The cDNA for real-time qPCR was synthesized from 1 µg of total RNA using a Verso cDNA kit (Thermo Scientific), with a 1:3 (v/v) blend of anchored oligo(dT) and random hexamers as primers, according to the manufacturer’s recommendations.

Real-time qPCR was performed on a CFX96 Touch Real-Time PCR Detection System (Bio-Rad Laboratories) in 0.2ml 96-well low-profile skirted plates using iTAQ Universal SYBR Green Supermix (Bio-Rad). The expression of *PDC2_E1α* was normalized to that of *RACK1* (GenBank accession no. XM_001698013) and the actin gene (GenBank accession no. XM_001699016), commonly used as housekeeping genes in *Chlamydomonas*. The primers and PCR programs used are listed in Supplementary Table S2. Gene expression was quantified by the ΔΔC_q_ method in CFX Manager™ Software (Bio-Rad). Real-time qPCR procedures and analyses were performed in accordance with MIQE guidelines (Bustin *et al.*, 2009).

### Pigment analysis

Pigments were extracted and analysed using a high-performance liquid chromatography system composed of a Prostar 240 solvent-delivery module, and a Prostar 330 photodiode array detector (Varian Analytical Instruments, Walnut Creek, CA, USA) and a C18 reverse-phase column (5 µm, 250mm Lichrosphere 100; Merck, Darmstadt, Germany) (Solovchenko *et al.*, 2013). Pigments were identified and quantified using pure pigment standards (Sigma-Aldrich, St Louis, MO, USA; Fluka, Taufkirchen, Germany).

### Photosynthetic activity estimation

The photosynthetic activity of the *C. reinhardtii* transformants was determined by: (i) fast Chl fluorescence transients; and (ii) oxygen evolution-based photosynthesis/irradiance (P-I) curves. The induction curves of Chl fluorescence were recorded using a Fluorpen FP100s portable Pulse Amplitude Modulated Fluorometer (Photon Systems Instruments, Drasov, Czech Republic) and analysed as described elsewhere (Strasser *et al.*, 2004; Solovchenko *et al.*, 2013). Non-photochemical quenching (NPQ) was calculated as NPQ=F_m_/F_m’_ – 1 (Maxwell and Johnson, 2000). Oxygen evolution by *C. reinhardtii* under an actinic irradiance increasing stepwise in the range 0–800 µE m^–2^ s^–1^ was monitored with a Clark-type oxygen electrode in the temperature-regulated chamber with a built-in computer-controlled LED light source (Chlorolab III; Hansatech, King’s Lynn, Norfolk, UK).

### Statistical analyses

To define whether observed differences between control and treatment cultures were significant, a two-tailed distribution Student’s *t*-test for two-sample unequal variance was used with an α less than 0.05. The data obtained for all replicates of two independent EV lines, 60 and 73, were combined and analysed together (designated EV).

## Results

### Bioinformatics survey for the *Chlamydomonas* PDH-E1α gene

The *C. reinhardtii* genome (http://www.phytozome.net/) was screened for candidate PDH subunit E1α-encoding genes for amiRNA silencing. A BLAST search was performed against an amino acid sequence of a plastid PDH subunit E1α isoform of *A. thaliana* (The Arabidopsis Information Resource, accession no. AT1G01090) as a query. An amino acid sequence with a high (>65%) similarity to the *Arabidopsis thaliana* protein and tentatively annotated as PDH E1α was found (PDC2_E1α; Cre02.g099850.t1.3). The nucleotide sequence of *PDC2_E1α* was selected for amiRNA silencing based on the results of the phylogenetic analysis, and a high identity of the deduced protein (60–70%) to homologous proteins from higher plants and cyanobacteria that are considered ancestors of chloroplasts (Supplementary Fig. S1 at *JXB* online). The BLAST search against the NCBI non-redundant protein database did not reveal a high similarity of the deduced protein to either plant or algal mitochondrial PDH E1α homologues, or to those from bacteria, which are commonly accepted as progenitors of mitochondria. Furthermore, another gene in the *C. reinhardtii* genome is annotated as encoding a mitochondrial PDH subunit E1α (PDC1; Cre07.g337650). A phylogenetic position similar to the putative chloroplast PDH subunit E1α was previously shown for the E2 subunit (DLA2) of the cpPDC of *C. reinhardtii* (Bohne *et al.*, 2013), whose chloroplast localization was confirmed experimentally. Nevertheless, it should be noted that the protein-localization prediction servers, such as TargetP and ChloroP (http://www.cbs.dtu.dk) (Emanuelsson *et al.*, 2000), as well as a new tool Predalgo (Tardif *et al.*, 2012), dedicated to algae, did not predict the chloroplast localization of the protein encoded by *PDC2_E1α*. However, the chloroplast localization was supported by Plant-mPLoc (http://www.csbio.sjtu.edu.cn/bioinf/plant-multi/) and MultiLoc2-LowRes (http://abi.inf.uni-tuebingen.de/Services/MultiLoc2), the latter taking into account phylogenetic and gene ontological terms. Further experimental evidence is required to confirm the plastid localization of the protein.

### Selection of the *pdh* silenced lines

**Fig. 1.**
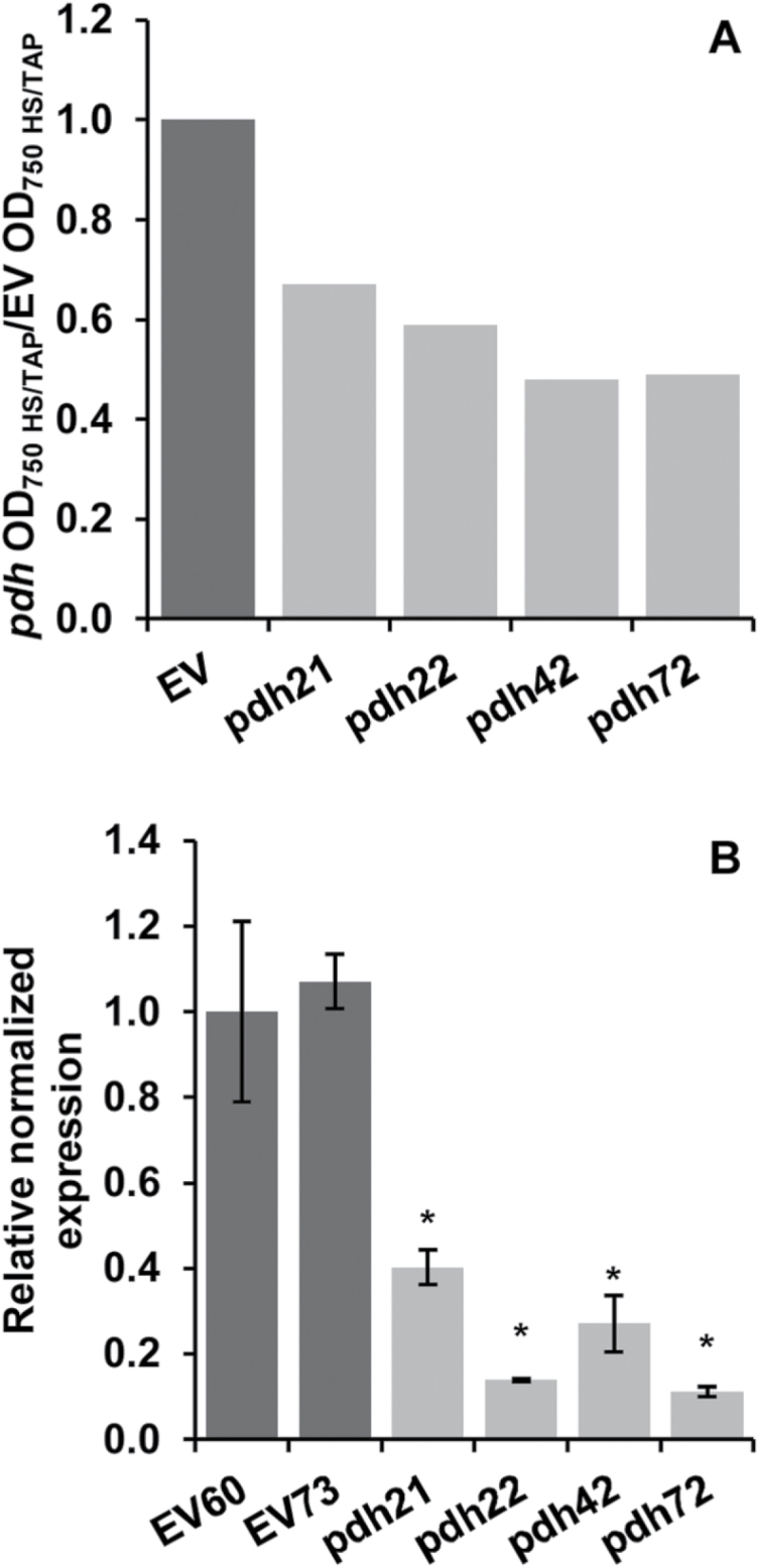
Selection of *C. reinhardtii* transformants. (A) OD of selected EV and *pdh* lines grown in HSM versus TAP medium. Dark grey bars, EV control lines; light grey bars, *pdh* lines. The average ratio of two different EV control lines is shown and was set to 1. (B) Expression of *PDC2_E1α* in the selected EV and *pdh* lines analysed by real-time qPCR. Statistical analysis was performed in Bio-Rad CFX Manager 3.0. Data are means±SD of three technical replicates. Asterisks indicate statistical differences between control and mutant lines at *P*<0.05 (Student’s *t*-test).

Assuming that downregulation of the *PDC2_E1α* gene would impair the growth of putative transformants more strongly under photoautotrophic conditions (in HSM) than under mixotrophic conditions (in TAP), we compared the OD_750_HSM:OD_750_TAP ratio of the *pdh* lines with that of the control EV lines, which was set to 1. Similar results were obtained when OD_680_ values were recorded (not shown). Selected lines demonstrating the lowest ratio (Fig. 1A) indeed showed a 60–90% reduction in *PDC2_E1α* expression, as confirmed by a real-time qPCR (Fig. 1B). Three mutants (*pdh21*, *pdh42*, and *pdh72*) were chosen for further characterization.

### Effect of PDC2_E1α silencing on *Chlamydomonas* growth parameters and total FA production in an nitrogen-replete medium under photoautotrophic and mixotrophic conditions

**Fig. 2.**
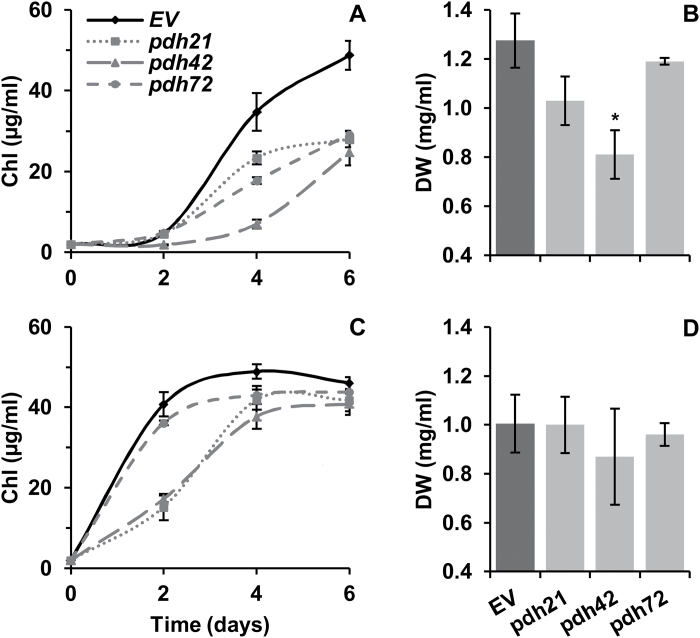
Effect of *PDC2_E1α* silencing on *C. reinhardtii* growth in HSM (A, B) and in TAP (C, D) medium. (A, C) Chl concentration. (B, D) Biomass concentration after 6 d of growth. Values shown for the *pdh* lines are means±SD of two biological and two technical replicates (*n*=4); EV values are means of two individual lines (*n*=8). Asterisks indicate significant differences between the EV and *pdh* lines at *P<*0.05 (Student’s *t*-test).

To investigate the impact of *PDC2_E1*α silencing on growth and total FA (TFA) production in the nitrogen-replete HSM and TAP media (designated HSM+N and TAP+N, respectively), *Chlamydomonas* cells were initially cultured in TAP+N and then transferred to either HSM+N or fresh TAP+N at an initial Chl concentration of 2 µg ml^–1^. During the duration of the experiment, lower Chl concentrations were determined in the *pdh* cultures in both HSM+N and TAP+N, compared with the EV lines (Fig. 2A, C). After a short initial lag, the EV cultures transferred to HSM+N resumed growth, whereas the growth of the *pdh* lines was substantially impaired, with a Chl content *~*40–50% lower than that in the EV lines by day 6. The final biomass of the *pdh* cultures in HSM+N was also lower than that of the controls, but there was no significant difference in the final DW in TAP+N (Fig. 2B, D). In TAP, the growth of the *pdh* lines was substantially retarded within the first 2 d, particularly in lines *pdh21* and *pdh72*, but the overall negative impact of *PDC2_E1α* silencing on the final Chl and DW contents was less pronounced under mixotrophic conditions (Fig. 2C).

**Fig. 3.**
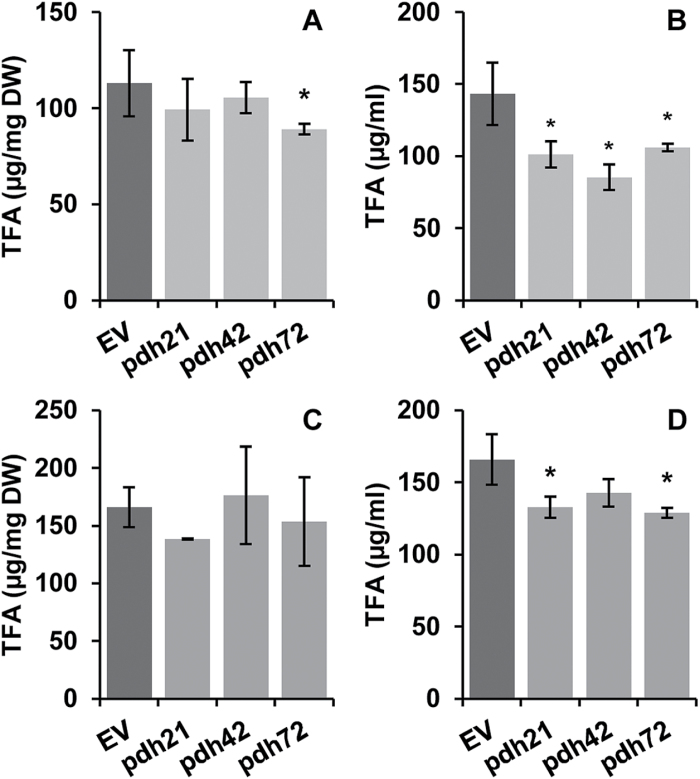
Effect of *PDC2_E1α* silencing on *C. reinhardtii* after 6 d of cultivation in HSM (A, B) and in TAP (C, D) medium. (A, C) TFA content of biomass. (B, D) Culture TFA content. Bars for the *pdh* lines represent means±SD of two biological and two technical replicates (*n*=4). EV values are means of two individual lines (*n*=8). Asterisks indicate significant differences between the EV and the *pdh* lines at *P<*0.05 (Student’s *t*-test).

After 6 d of photoautotrophic growth, the TFA content of the biomass was about 20% lower (*pdh72*) or similar to that of the EVs (Fig. 3A). Notably, the difference in TFA production between EV and *pdh* transformants was more evident when expressed in terms of volumetric FA content (µg ml^–1^), which accounts for both the culture DW content and the TFA content of the biomass (Fig. 3B). Given the substantial decrease in their final DW, the *pdh* mutants demonstrated *~*25–40% less volumetric TFA production than the control lines (Fig. 3B).

A similar but less prominent effect of *PDC2_E1α* silencing on TFA accumulation (Fig. 3C, D) and FA composition (Supplementary Fig. S2 at *JXB* online) was observed under mixotrophic conditions, in TAP+N. The volumetric content of TFA in the cultures of the *pdh* mutants was about 15–25% lower than that in the EV line cultures (Fig. 3D).

### Effect of PDC2_E1α silencing on *Chlamydomonas* biomass and total FA production in an nitrogen-depleted medium under photoautotrophic and mixotrophic conditions

**Fig. 4.**
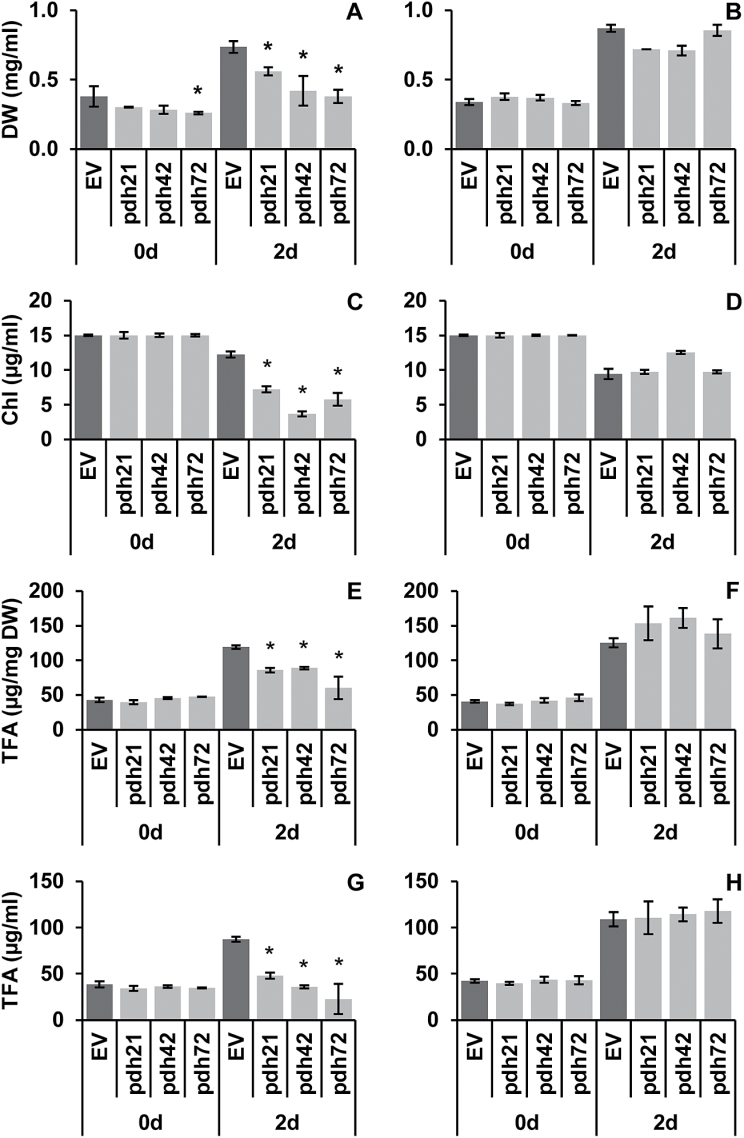
Effect of *PDC2_E1α* silencing on *C. reinhardtii* after 2 d of cultivation under nitrogen starvation in HSM-N (A, C, E, G) and in TAP-N (B, D, F, H) medium. (A, B) Biomass (DW) content. (C, D) Culture Chl content. (E, F) TFA content of biomass. (G, H) Culture TFA content. Values represent means±SD of *n*=6 for *pdh* lines (three biological and two technical replicates) and *n*=12 for EV control lines (joint results of two different control lines). Differences between controls and mutants are significant at *P<*0.05 (Student’s *t*-test).

Conditions of nitrogen starvation are generally associated, in microalgal cultures, with a decline in the per cell and per culture volume contents of Chl due to gradual chloroplast dismantling (Goodson *et al.*, 2011; Siaut *et al.*, 2011). To avoid the strong autophagous response common to *Chlamydomonas* under nitrogen starvation (Perez-Perez *et al.*, 2010), short-term experiments (lasting 48h) were performed. Three selected *pdh* lines demonstrated a stronger reduction in culture Chl content than the controls (Fig. 4C) cultivated in HSM-N, indicating an accelerated loss of photosynthetic pigments by the culture. The overall reduction in culture Chl content for the *pdh* lines was significantly more pronounced, constituting 40–70% of that in the EV cultures. Cell biomass (DW) production in the mutant cultures was also reduced, amounting to 51, 57, and 76% of the DW of the control lines for *pdh72*, *pdh42*, and *pdh21*, respectively (Fig. 4A). Due to a simultaneous decrease in both parameters, the Chl content of the biomass was not significantly altered compared with the EV lines, with the exception of line *pdh42* (not shown). In contrast, the downregulation of *PDC2_E1α* did not substantially alter either the culture Chl or the DW contents of *Chlamydomonas* in TAP-N (Fig. 4B, D), but some decrease in biomass production was noted in the lines *pdh21* and *pdh42.*

Unlike in TAP-N, where there were no obvious differences in TFA production between the different lines (Fig. 4F, H), all *pdh* lines demonstrated severe impairment of TFA production in HSM-N (Fig. 4E, G). After 2 d of nitrogen starvation, the biomass content of the TFA was 25–50% lower in the *pdh* lines relative to the EV lines in this medium (Fig. 4E). Even more marked differences between the silenced and the control lines were observed in the volumetric content of TFA due to the cumulative effect of *PDC2_E1α* knockdown on biomass production and the TFA content of DW; the *pdh* lines produced 45–75% less TFA than the EV (Fig. 4G). Notably, TFA production was almost entirely abolished in the nitrogen-starved photoautotrophic cultures of the *pdh* lines 42 and 72 compared with time 0 (Fig. 4G), while it was unaltered in the mixotrophic cultures of the *pdh* lines in comparison with the control (Fig. 4H).

**Fig. 5.**
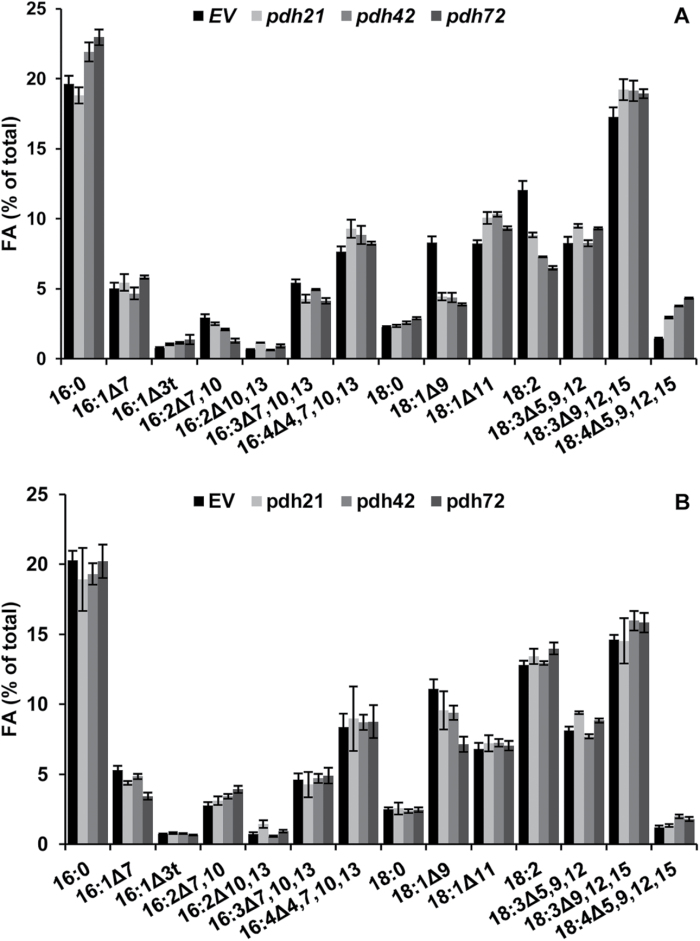
Effect of *PDC2_E1α* silencing on FA composition of *C. reinhardtii* after 2 d of nitrogen starvation in HSM-N (A) and in TAP-N (B) medium. Bars show means±SD of three biological and two technical replicates (*n*=6). EV bars represent means of two individual lines (*n*=12).

The downregulation of *PDC2_E1α* led to substantial changes in the FA profile of total lipids under photoautotrophic conditions, specifically to a *~*50% decrease in the proportion of oleic acid (OA, 18:1^Δ9^) in the *pdh* lines relative to the EV lines after 2 d of nitrogen starvation (Fig. 5A). A decline in the relative proportions of OA, as well as linoleic acid (18:2^Δ9,12^), was accompanied by an increase in the proportions of the C18 polyunsaturated FA (PUFA) α-linolenic acid (ALA; 18:3^∆9,12,15^) and 18:4^∆5,9,12,15^ (Fig. 5A). Marked alterations were observed in the percentages of C16 PUFA; a decrease in the proportion of 16:3^∆7,10,13^ was accompanied by a concomitant increase in that of 16:4^∆4,7,10,13^; 16:0 increased in the *pdh* lines 42 and 72.

Remarkably, less substantial changes in the FA profile were found between the *pdh* and the EV lines under mixotrophic conditions (Fig. 5B), when acetate was added to the medium along with CO_2_.

### PDC2_E1α silencing decreases TAG production and affects the FA composition of TAG under phototrophic conditions combined with nitrogen deprivation

**Fig. 6.**
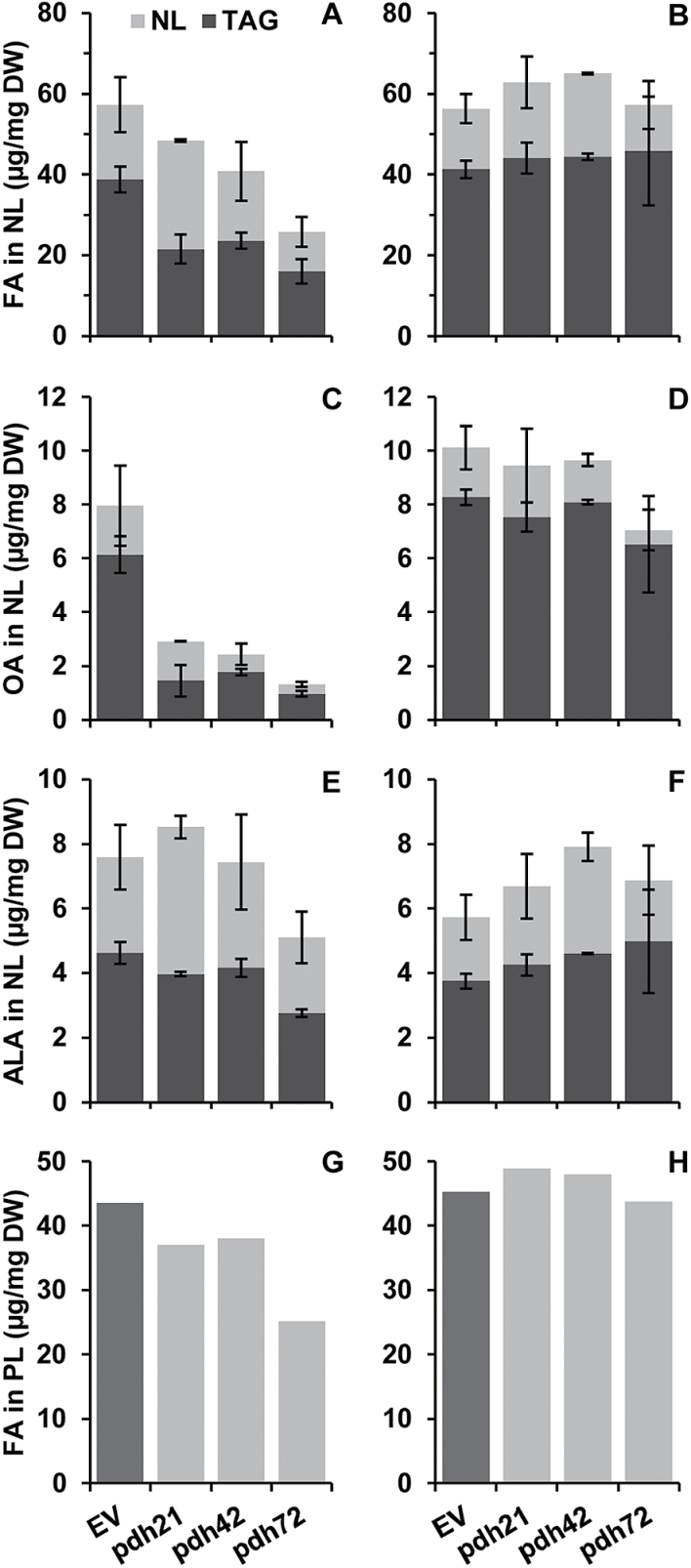
Effect of *PDC2_E1α* silencing on FA distribution in NLs, TAG (A–F), and in PLs (G, H) of *C. reinhardtii* after 2 d of nitrogen starvation in HSM-N (A, C, E, G) and in TAP-N (B, D, F, H) medium. The contribution of TAG total FA, OA, and ALA to NLs is shown in dark grey (A)–(F). Values shown (except for PL in G and H) are the means±SD of two technical replicates from pooled samples of three biological replicates. Analysis of FA content in PL was performed from the pooled samples with two replicates. EV bars represent means of two individual lines.

To elucidate whether the observed alterations in TFA content and FA composition were associated with alterations in the distribution of individual lipid classes and changes in their FA profiles, we further separated the total lipid extracts of the biomass samples, collected under nitrogen starvation, into NL and PL fractions to isolate TAG and individual membrane lipids, respectively. In HSM-N, the total acyl group content in the NL fraction (expressed as FA content) was significantly lower in the *pdh* lines than in the controls (Fig. 6A), with the most drastic decrease featured in the line *pdh72*. Remarkably, TAG content in the biomass of the *pdh* lines constituted only *~*45–60% of the TAG content in the EV lines (Fig. 6A). In line with these data, microscopic observations of the cells stained with the lipophilic dye Nile red revealed a strong decrease in the number of lipid droplets in the cells of all three *pdh* lines (not shown).

**Fig. 7.**
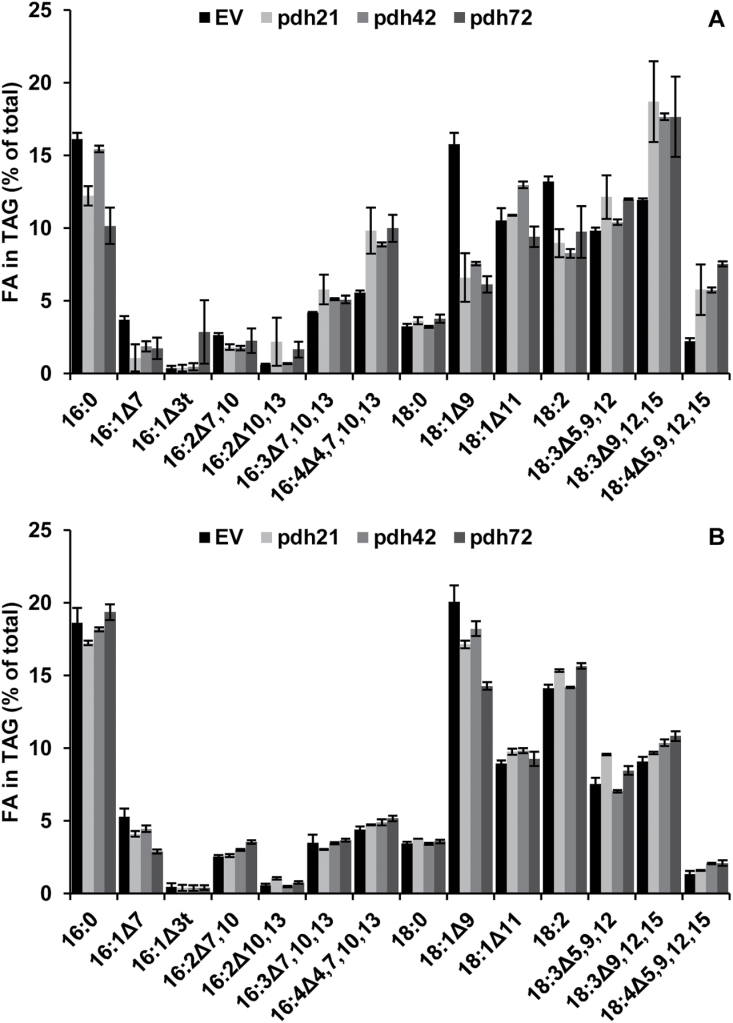
Effect of *PDC2_E1α* silencing on FA composition of TAG of *C. reinhardtii* after 2 d of nitrogen starvation in HSM-N (A) and in TAP-N (B) medium. Bars show the means±SD of two technical replicates from pooled samples of three biological replicates. EV bars represent means of two individual lines.

The reduced TAG production in the cells of the *pdh* lines in HSM-N was associated with substantial alterations in the FA profile of TAG (Fig. 7A). The most drastic effect was determined on the relative percentage of 18:1^∆9^, which accounted for only 50–60% of that in the controls after 2 d of nitrogen starvation. Accordingly, *~*60–85% and 70–85% reductions in the 18:1^∆9^ content (µg mg^–1^) allocated to NL and TAG, respectively, were found in the *pdh* lines compared with the controls (Fig. 6C). Similarly, the percentage of another product of *de novo* FA synthesis, palmitic acid (16:0), decreased in the TAG of the *pdh* lines 21 and 72, along with *~*40–75% reductions in its content deposited in the TAG of three *pdh* lines (not shown). The consistent decreases in the proportions of 18:1^∆9^ and 18:2^Δ9,12^ occurred concomitantly with the increases in those of 16:4^Δ4,7,10,13^ and 18:3^Δ9,12,15^ (Fig. 7A), the latter two being the major acyl constituents of chloroplast galactolipids in *Chlamydomonas* (Giroud *et al.*, 1988; Sakurai *et al.*, 2014). Accordingly, the absolute contents of ALA (18:3^Δ9,12,15^) deposited in TAG did not decrease substantially in the *pdh* lines (Fig. 6E). These data may indicate that the flux of chloroplast-resident acyl groups into TAG (Li *et al.*, 2012) was not altered in the *pdh* lines but rather the *de novo* TAG biosynthesis was affected that is reliant on 16:0 and 18:1^∆9^ production in the chloroplast. Again, the strongest impact of *PDC2_E1α* silencing was observed in line *pdh*72. In addition, a 2.5- to 3.5-fold increase was determined in the percentage of 18:4^Δ5,9,12,15^ in the FA profile of TAG in the *pdh* lines (Fig. 7A).

In contrast to photoautotrophic conditions, comparable FA contents in NL and TAG were determined in the mutant and control nitrogen-depleted cultures (Fig. 6B) in TAP-N; furthermore, overall alterations in the FA profile of TAG were less marked in the *pdh* lines (Fig. 7B). The percentage of OA in the FA profile of TAG in the *pdh* lines amounted to 70–90% of that found in the EV; the line *pdh*72 had the lowest OA content (Fig. 7B).

### PDC2_E1α silencing affects the FA composition of membrane glycerolipids under phototrophic conditions combined with nitrogen deprivation

Whereas the silencing of *PDC2_E1α* had a crucial effect on TAG formation in HSM-N, it had a lesser effect on PL content (Fig. 6G), supporting the assumption that an alleviated acetyl-CoA supply mainly affected the induced storage lipid biosynthesis driven by nitrogen starvation. It should be noted, however, that *pdh72*, featuring the strongest decrease in NL, also showed the biggest decrease in PL content, implying a negative effect on structural membrane lipids.

The strongest impact of *PDC2_E1α* silencing was found on the relative content of the chloroplast galactolipid (MGDG), whose relative percentage of PL was markedly reduced in all *pdh* lines. The proportion of digalactosyldiacylglycerol (DGDG) did not seem to be substantially altered, indicating a decreased MGDG:DGDG ratio in the cells with reduced expression of *PDC2_E1α* (Supplementary Fig. S3 at *JXB* online). The percentage of the major extraplastidial betaine lipid diacylglyceryl-*N*,*N*,*N*-trimethylhomoserine (DGTS) in the *pdh* lines did not change substantially compared with the EV control.

The individual lipid classes of *Chlamydomonas* possess distinct and characteristic FA signatures (Giroud *et al.*, 1988; Sakurai *et al.*, 2014), and they demonstrated specific alterations in the *pdh* lines (Supplementary Fig. S4 at *JXB* online). A clear trend towards more unsaturated acyl groups was observed in major lipid classes. MGDG displayed substantially higher percentages of 16:4^Δ4,7,10,13^ and 18:3^Δ9,12,15^ in all analysed *pdh* lines compared with the control. DGDG of the *pdh* lines showed a clear trend towards more unsaturated C18 FA species in comparison with the control. The chloroplast phospholipid phosphatidylglycerol featured an increased percentage of its unique FA 16:1^Δ3t^ in the *pdh* lines (except for the line 42) against the background of the reduced proportion of 16:0, as well as a trend of an increasing percentage of 18:3^Δ9,12,15^, similar to galactolipids. The most noticeable alterations in the FA profile of DGTS accounted for the increased proportion of 16:0 and the decreased proportion of 18:2^Δ9,12^. Among the two Δ5 C18 PUFA, 18:3^Δ5,9,12^ and 18:4^Δ5,9,12,15^, which are particularly enriched in DGTS, a noticeable increase was documented in the proportion of 18:4^Δ5,9,12,15^ in all *pdh* lines. The sulfolipid sulfoquinovosyl diacylglycerol (SQDG) and the phospholipid phosphatidylinositol contained high proportions of 18:1^Δ11^. Notably, as in SQDG and phosphatidylglycerol, the proportion of 18:1^Δ11^ in DGTS was higher than that of 18:1^Δ9^, with a slight increase in the former in the silenced lines. The identity of 18:1^Δ11^ was confirmed by the GC mass spectrometry of 4,4-dimethyloxazoline derivatives (not shown).

### Effects of PDC2_E1α silencing on carotenoid profiles and photosynthetic activity

Next, we investigated the composition of individual carotenoids in the transformed cells of *C. reinhardtii*. The carotenoid profiles of all of the studied transformants grown in TAP were similar (Supplementary Fig. S5 at *JXB* online), comprising the carotenoids typical of the wild-type *C. reinhardtii* (Lohr *et al.*, 2005). Cultivation in HSM introduced no changes to the qualitative carotenoid composition regardless of the transformant line or nitrogen availability. Only a small increase in the de-epoxidation state of the violaxanthin cycle [violaxanthin, antheraxanthin, zeaxanthin (VAZ)] was detected after 2 d of nitrogen starvation; the magnitude of this increase was roughly similar in all transformant lines (Supplementary Fig. S6A at *JXB* online), as was the total size of the VAZ pool (Supplementary Fig. S6B).

All of the studied transformant lines (EV and *pdh*) grown in TAP were characterized by a uniformly high maximum quantum efficiency of PSII (estimated as F_v_/F_m_; Table 1), an indication of high photosynthetic activity. Cultivation in HSM brought about a small but significant decrease in F_v_/F_m_, which was more pronounced in the *pdh* lines than in the EV (Table 1). Nitrogen starvation induced an apparent and more profound decline in PSII efficiency of the *pdh* lines, whereas the EV lines retained F_v_/F_m_ at a level typical of nitrogen-sufficient cultures. A similar pattern of changes was found in the slope of the linear part of the P-I curves and the maximum rate of photosynthesis (*P*_max_; Supplementary Fig. S7 at *JXB* online), recorded via oxygen evolution. It should be noted that, under mixotrophic conditions, *P*_max_ was slightly higher in the *pdh* lines than in the EV. The considerable increase in F_0_ against the background of a decrease in Chl content (Supplementary Table S4 at *JXB* online), observed in the *pdh* lines under nitrogen starvation may suggest a certain degree of stress-induced disorders in the reaction centres of the cells.

**Table 1.** Condition of photosynthetic apparatus of C. reinhardtii transformants grown in TAP+N, HSM+N for 6 d and in HSM-N for 2 dF_v_/F_m_, maximum potential quantum yield of the PSII; M_0_, changes in the initial slope of the fast chlorophyll fluorescence transient (indicative of Q_A_ reduction; for the details of the calculations, see Supplementary Table S3). Values shown are the means±SD of three biological replicates. EV values represent the means of two individual lines.

Strain	Medium	F_v_/F_m_	M_0_
EV	TAP+N	0.74±0.00	0.98±0.02
	HSM+N	0.75±0.01	1.05±0.13
	HSM-N	0.73±0.01	1.36±0.05
*pdh21*	TAP+N	0.72±0.01	0.95±0.03
	HSM+N	0.71±0.01	1.13±0.10
	HSM-N	0.65±0.01	1.65±0.04
*pdh42*	TAP+N	0.73±0.00	1.03±0.01
	HSM+N	0.71±0.00	1.25±0.04
	HSM-N	0.66±0.01	1.76±0.04
*pdh72*	TAP+N	0.72±0.01	1.01±0.01
	HSM+N	0.64±0.01	1.57±0.05
	HSM-N	0.57±0.01	2.10±0.05

To obtain additional insight into the responses of the photosynthetic apparatus of the generated *C. reinhardtii* transformants to cultivation in nitrogen-replete and nitrogen-depleted mineral media, the analysis of fast Chl fluorescence transients (JIP test) was employed (Supplementary Table S3 at *JXB* online; see also Strasser *et al.*, 2004). Notably, little, if any, difference was found in the condition of the photosynthetic apparatus in the *pdh* transformant lines grown in TAP in comparison with the EV (Table 1). On the other hand, transfer into HSM+N resulted in a decrease in the maximum Chl fluorescence level, relative to F_0_ (Supplementary Fig. S8 at *JXB* online) and in an increase in the reduction of the primary acceptor, Q_A_ (Table 1), even in the dark-adapted samples. Accordingly, Stern–Volmer coefficients of NPQ in these cultures exhibited a very low response to increased irradiances (not shown). Under nitrogen starvation, these effects were much more pronounced in the *pdh* cells, whereas they were less conspicuous in the EV lines (Table 1 and Supplementary Table S4). The recorded changes in the Chl fluorescence parameters are evidence of the higher degree of electron carrier reduction in the chloroplast electron transport chain in the *pdh* cells under photoautotrophic conditions.

## Discussion

The present study focused on elucidating the role of cpPDC in the supply of acetyl-CoA for *de novo* FA synthesis under conditions favouring TAG accumulation in the cells of photoautotrophic microalgae. As a model, we used the green microalga *C. reinhardtii*, which exhibits flexible responses in its central carbon and lipid metabolism to environmental perturbations, including nitrogen starvation (Merchant *et al.*, 2012).

RNAi approaches have been widely employed in *C. reinhardtii*, recently also encompassing lipid metabolism (Moellering and Benning, 2010; Cerutti *et al.*, 2011; Williams *et al.*, 2011; Bohne *et al.*, 2013). In the current study, *C. reinhardtii* mutants with reduced *PDC2_E1α* expression were generated and selected based on their impaired capability of photoautotrophic growth. The ability of the knockdown lines to produce TAG appeared to be severely impaired in photoautotrophic conditions when photosynthetic carbon fixation provides precursors for the synthesis of storage products. It is worth noting that RNAi-mediated deficiency in DLA2, an E2 subunit of the cpPDC from *C. reinhardtii*, did not cause any apparent qualitative effect on the composition of membrane glycerolipids under phototrophic nitrogen-replete conditions (Bohne *et al.*, 2013). However, the impact of *DLA2* knockdown on the storage lipid TAG accumulation under different carbon regimes was not examined.

### Reduced expression of PDC2_E1α underscores the essential role of cpPDC in the carbon supply for FA synthesis from photosynthetically fixed carbon

PDH E1α downregulation did not show a strong effect on Chl, biomass, and TAG production under mixotrophic conditions, indicating that photosynthetically fixed carbon is less important in the presence of acetate. Our results firmly support the role of acetate as a source of acetyl-CoA for FA synthesis under mixotrophic conditions in *Chlamydomonas* in agreement with other studies (Work *et al.*, 2010; Fan *et al.*, 2012; Goodenough *et al.* 2014).

Under photoautotrophic conditions, the accelerated loss of photosynthetic pigments and reduced biomass production in the cultures of the knockdown *pdh* lines occurred under nitrogen starvation concomitantly with a drastic reduction in the content of TAG. Hence, the provision of acetyl-CoA through cpPDC activity to the initial and committed step of *de novo* FA synthesis, namely to acetyl-CoA carboxylase, seems to be indispensable for *Chlamydomonas* grown photoautotrophically. In addition, it should be noted that carbon precursors for FA synthesis might be provided via starch catabolism in the chloroplasts, as the synthesis and breakdown of starch occur in the same compartment as FA synthesis and precede TAG formation in *Chlamydomonas* (Fan *et al.*, 2012). However, the metabolic interaction between the two major storage products is still under debate (Work *et al.*, 2010; Siaut *et al.*, 2011; Wase *et al.*, 2014), and is mostly studied under conditions of mixotrophy. Regarding the origin of pyruvate in *Chlamydomonas*, lacking the chloroplast pyruvate kinase (Klein 1986; Terashima *et al.*, 2011), the malic enzyme may play the role of the main pyruvate supplier for cpPDC activity. Alternatively, pyruvate can be shuttled to the chloroplast via dedicated transporters (Facchinelli and Weber, 2011; Li *et al.*, 2014). It is noteworthy that recent ‘omic’ studies have documented an elevated level of citrate in nitrogen-starved cells of *Chlamydomonas*, as well as commensurate alterations in the abundances of enzymes mediating its synthesis and utilization, implying a potential role of this central metabolite as a carrier of acetyl groups for induced storage lipid accumulation (Wase *et al.*, 2014). However, the mechanisms that may permit the bulky molecule of acetyl-CoA to enter the plastid remain elusive and warrant further investigations. We assume that conditions of nitrogen starvation lead to the partial endomembrane dismantling that might affect chloroplast membrane integrity and alter trafficking of central carbon metabolites into the plastid.

### Characteristic changes in FA composition of individual glycerolipids indicate membrane lipids as the major source of FA for TAG in the *pdh* lines under conditions of photoautotrophy

Comparing the FA composition of TAG with that of polar lipids in the *pdh* lines in HSM-N, one can correlate the apparent similarity in the increased proportion of 16:4^Δ4,7,10,13^ and 18:3^Δ9,12,15^ and the decreased percentages of the less unsaturated precursors of these PUFA, in both TAG and galactolipids, specifically in MGDG. Overall alterations in the FA composition of TAG indicated a prevalence of molecular species that were assembled from diacylglycerol precursors of the chloroplast origin with a characteristic arrangement of C16 and C18 PUFA at the *sn*-2 and *sn*-1 positions of the glycerol moiety, respectively (Fan *et al.*, 2011; Li *et al.*, 2012). Another diagnostic feature of the TAG in the silenced lines—the decreased proportion and content of 18:1^Δ9^—seems to result from the suppression of the *de novo* FA synthesis supplying this FA for TAG assembly. Similar alterations in the FA composition of TAG were described in the *pdg1* (plastid galactoglycerolipid degradation) mutant of *C. reinhardtii* impaired in OA channelling from the *de novo* synthetized MGDG (Li *et al.*, 2012). However, in contrast to *PDC2_E1α* silencing, a *pgd1* mutation led to drastically decreased TAG content under mixotrophic conditions (Li *et al.*, 2012). Given that, in the TAG of *Chlamydomonas*, OA is predominantly localized to the *sn*-1/*sn*-3 positions (Fan *et al.*, 2011; Li *et al.*, 2012), it is likely that an impaired acetyl-CoA supply and reduced FA synthesis in the *pdh* mutants affected the flux of the *de novo*-produced OA onto those positions of TAG.

Notably, the mutation in *PGD1* did not lead to substantial changes in the FA composition of membrane lipids under conditions of nitrogen starvation (Li *et al.*, 2012), in contrast to the clear impact of *PDC2_E1α* knockdown on the FA composition of membrane lipids. The FA profile of the chloroplast lipids of the *pdh* lines revealed the seemingly higher level of chloroplast lipid unsaturation, and particularly of MGDG, probably retaining the FA profile of the initial non-stressed cells, which can be explained by the restricted *de novo* supply of FAs.

Our results are in agreement with the suggested model of cooperation of multiple pathways and cellular compartments in TAG assembly in *Chlamydomona*s (Fan *et al.*, 2011; Li *et al.* 2012; Liu and Benning, 2013; Sakurai *et al.*, 2014). The contribution of different pathways and compartments to the supply of acyl groups for TAG formation under nitrogen depletion seems to vary depending on the carbon regime. Notably, FA moieties characteristic of membrane lipids were prevalent in the downsized TAG of the *pdh* lines under conditions of phototrophy. The significance of the supply of unsaturated acyl moieties for TAG assembly from both plastidic and extraplastidial lipids has been inferred previously (Li *et al.*, 2012; Sakurai *et al.*, 2014), but the exact mechanisms require in-depth investigation.

Intensive membrane dismantling and increased transcript abundance of genes encoding numerous lipases and autophagy-associated genes is the characteristic *Chlamydomonas* response to nitrogen scarcity (Perez-Perez *et al.*, 2010; Goodson *et al.*, 2011; Boyle *et al.*, 2012; Blaby *et al.*, 2013; Goodenough *et al.*, 2014). Notably, a stronger effect on chloroplast breakdown in the WT-12 *nit* ^–^ *C. reinhardtii* was observed under photoautotrophic conditions, while acetate addition reduced autophagy in long-term experiments (Davey *et al.*, 2014), underscoring the alleviating effect of the organic carbon source due to its use in FA and energy production. A substantial reduction in the proportion of MGDG was documented in the wild-type *C. reinhardtii* strain CC1010 under photoautotrophic nitrogen-deficient conditions (Sakurai *et al.*, 2014), whereas two other major lipids, DGDG and DGTS, sustained higher relative levels in the starved cells in line with our data on individual lipid class distribution. A reduced relative content of MGDG was documented in the *pdh* lines under nitrogen starvation compared with the controls in accordance with our suggestion that chloroplast lipid turnover is a major source of acyl groups for the residual TAG formation in the *pdh* lines under photoautotrophy. It is likely that the altered MDGD:DGDG ratio in the *pdh* lines that is crucial for a proper functioning of photosynthetic complexes in thylakoids could have a negative impact on the photosynthetic capacity of the knockdown lines.

### TAG biosynthesis primed by cpPDC is important for nitrogen-stress acclimation under photoautotrophic conditions

Our results imply the deleterious effect of *PDC2_E1α* downregulation on the Chl content and photosynthetic capacity of *Chlamydomonas* under conditions of photoautotrophy. The impaired-growth phenotype, displaying reduced Chl culture and biomass content in the *pdh* mutants under nitrogen starvation, was comparable to the chlorotic phenotype observed in a *pgd1* mutant of *Chlamydomonas* (Li *et al.*, 2012). It was hypothesized that the reduced survival of the *pgd1* mutant under nitrogen deprivation arises from cell damage by reactive oxygen species, whose level increased when photosynthesis was still functional but TAG accumulation, an important sink of the carbon excessively fixed during nitrogen starvation, was compromised (Li *et al.*, 2012). The significance of FA synthesis, for the purposes of TAG overproduction in microalgae under conditions of an augmented carbon:nitrogen ratio, was indeed related to the prevention of photooxidative damage (Hu *et al.*, 2008; Li *et al.*, 2012; Solovchenko, 2012).

The reduced ability of *pdh* lines for TAG production might have led to an impaired sink capacity for photosynthates and hence to a decline in photosynthetic activity. According to the analysis of electron transport at the acceptor side of PSII, this impairment of photosynthetic activity could have been caused by a sustained over-reduction of electron carriers in the photosynthetic electron transport chain. It is unlikely that *PDC2_E1α* silencing exerted a direct detrimental effect on the photosynthetic machinery since the photosynthesis in the transformed lines appeared to be undisturbed in the TAP-grown cells in which the sink furnished by TAG biosynthesis was less impaired. It should also be emphasized that the degree of decline in TAG biosynthesis correlated well with the decline in various photosynthetic parameters. Thus, the most pronounced effects, on both TAG and the rate of photosynthesis, were recorded in the line *pdh72* (Table 1, Supplementary Table S4). In contrast to the *pgd1* mutant, no strong effect on TAG production was determined in the *pdh* lines in the presence of acetate, underscoring the essential role of cpPDC in the acetyl-CoA supply under photoautotrophic conditions. Indeed, neither pigment profile nor photosynthetic activity was affected in the *pdh* lines under mixotrophic conditions. It seems that when acetyl-CoA supply is not impaired, photosynthates are consumed efficiently, in the reactions of FA synthesis and consequent TAG biosynthesis, by the *pdh* lines to the same extent as by the EV lines, developing a phenotype that is similar to the controls.

To conclude, the results obtained in the present research suggest the dependence of *de novo* FA synthesis and TAG biosynthesis in photoautotrophic *Chlamydomonas* cells on the transcriptional activation of cpPDC. Our results strongly support a crucial role for cpPDC in supplying acetyl-CoA for *de novo* FA synthesis under photoautotrophic conditions; hence, the reduced expression of the *E1α* subunit of PDH is detrimental to storage lipid production and to the overall acclimation of the cells to nitrogen starvation. Further studies may delineate the effects of the restricted acetyl-CoA production on transcriptome reprogramming and alterations in the central carbon metabolism. In addition, further exploitation of cpPDC-encoding gene overexpression to enhance TAG production in non-model oleaginous microalgae under photoautotrophic conditions should be considered.

## Supplementary data

Supplementary data are available at *JXB* online.

Supplementary Table S1. 90-bp oligonucleotides used for pChlami2-amipdh vector construction for gene silencing.

Supplementary Table S2. On-colony PCR and real-time qPCR primers, and conditions of qPCR.

Supplementary Table S3. Characteristic points of OJIP and derivation of the selected JIP test parameters used in this work.

Supplementary Table S4. Characteristic parameters of chlorophyll fluorescence in the cells of *pdh* and EV lines of *C. reinhardtii.*

Supplementary Fig. S1. Phylogeny reconstruction of plastid and mitochondrial isoforms of pyruvate dehydrogenase (PDH) E1α subunit from plant, algal, and bacterial species.

Supplementary Fig. S2. Effect of *PDC2_E1α* silencing on FA composition of *C. reinhardtii* after 6 d cultivation.

Supplementary Fig. S3. Effect of *PDC2_E1α* silencing on relative proportion of major polar lipid classes in EV and *pdh* lines after 2 d of nitrogen starvation under photoautotrophic conditions.

Supplementary Fig. S4. Fatty acid composition of individual polar lipids after 2 d of nitrogen starvation under photoautotrophic conditions.

Supplementary Fig. S5. Carotenoid composition of the *pdh* and EV lines of *C. reinhardtii* cells at the onset and after 2 d of nitrogen starvation under photoautotrophic conditions.

Supplementary Fig. S6. De-epoxidation state (A) of the violaxanthin cycle and violaxanthin cycle pigment pool size (B) of the *pdh* and EV lines of *C. reinhardtii* cells at the onset and after 2 d of nitrogen starvation under photoautotrophic conditions.

Supplementary Fig. S7. Photosynthetic activity of the *pdh* and EV lines of *C. reinhardtii* cells at the onset of the experiment, and after 6 d in HSM+N and 2 d in HSM-N as manifested by P-I curves recorded via oxygen evolution.

Supplementary Fig. S8. Typical curves of chlorophyll fluorescence induction (OJIP) recorded in the initial culture of the *pdh* and EV lines of *C. reinhardtii* grown in TAP (A) and after 2 d of nitrogen starvation in HSM-N (B).

Supplementary Data

## Abbreviations:

ALA: α-linolenic acid
ACS: acetyl-CoA synthase
amiRNA: artificial microRNA
Chl: chlorophyll
cp: chloroplast
DGDG: digalactosyldiacylglycerol
DGTS: diacylglyceryl-*N,N,N*-trimethylhomoserine
DLA2: dihydrolipoamide acetyltransferase
DW: dry weight
EV: empty vector
FA: fatty acid
GC: gas chromatography
HSM: high-salt minimal media
MGDG: monogalactosyldiacylglycerol
NL: neutral lipids
NPQ: non-photochemical quenching
OA: oleic acid
PDC: pyruvate dehydrogenase complex
PDH: pyruvate dehydrogenase
PEP: phosphoenolpyruvate
P-I: photosynthesis/irradiance
PL: polar lipids
PSII: photosystem II
PUFA: polyunsaturated fatty acid
qPCR: quantitative PCR
SQDG: sulfoquinovosyl diacylglycerol
TAG: triacylglycerol
TAP: Tris-acetate-phosphate medium
TFA: total fatty acids
VAZ: violaxanthin, antheraxanthin, zeaxanthin.
