# Supplementary material for: Downregulation of a putative plastid PDC E1α subunit impairs photosynthetic activity and triacylglycerol accumulation in nitrogen-starved photoautotrophic Chlamydomonas reinhardtii
Source: J Exp Bot. 2014 Sep 10;65(22):6563–76. doi: 10.1093/jxb/eru374 (PMC4246187; doi:10.1093/jxb/eru374)
Supplement: Supplementary Data [file supp_65_22_6563__index.html]

Downregulation of a putative plastid PDC E1α subunit impairs photosynthetic activity and triacylglycerol accumulation in nitrogen-starved photoautotrophic Chlamydomonas reinhardtii — Supplementary Data 

# Downregulation of a putative plastid PDC E1α subunit impairs photosynthetic activity and triacylglycerol accumulation in nitrogen-starved photoautotrophic *Chlamydomonas reinhardtii*

## Supplementary Data

Data files

**Files in this Data Supplement:**

- Supplementary Data - Supplementary Data
