# Supplementary material for: Downregulation of a putative plastid PDC E1α subunit impairs photosynthetic activity and triacylglycerol accumulation in nitrogen-starved photoautotrophic Chlamydomonas reinhardtii
Source: J Exp Bot. 2014 Sep 10;65(22):6563–76. doi: 10.1093/jxb/eru374 (PMC4246187; doi:10.1093/jxb/eru374)
Supplement: Supplementary Data [file supp_eru374_Supplementary_Data_JXB_after_revision_corrected.pdf]

Supplementary Data

Table S1. 90-bp Oligonucleotides used for pChlami2-ami*pdh* vector construction for gene silencing.

ami-PDC2_E1α-F	5' - ctagtATGTGCGCCCAGATGTTCTAAtctcgctgatcggcaccatgggggtggtggtgatcagcgctaTTAGTACATCTGGGCGCACATg -3'
ami-PDC2_E1α-R	5' - ctagcATGTGCGCCCAGATGTACTAAtagcgctgatcaccaccacccccatggtgccgatcagcgagaTTAGAACATCTGGGCGCACATa -3'

Table S2. On-colony PCR and Real-Time qPCR Primers.

pChlamiRNA2-F	5'-TGTGGGACTTCAACCGCAGC-3'
pChlamiRNA2-R	5'-GCCCGCCAAATCAGTCCTGT-3'
Act-Cr-RT-F	5'-ATCTGGCACCACACCTTCTTCAAC-3'
Act-Cr-RT-R	5'-GAAGGTCTCGAACATGATCTGGGT-3'
RACK1-Cr-RT-F	5'-CACCACGGCTACGTCAACACC-3'
RACK1-Cr-RT-R	5'-AGAAGCACAGGCAGTGGATGAC-3'
PDH-Cr-RT-F	5'-AGTTCTACGAGTCGCTCAACATGG-3'
PDH-Cr-RT-R	5'-TCTTGTAGATGTGCGGATCCTGGT-3'

RT-qPCR protocol:

95.0 C for 5:00

95.0 C for 0:10

60.0 C for 0:20

72.0 C for 0:20 + Plate Read

GOTO 2, 39 more times

Melt Curve 55.0 to 95.0 C, increment 0.5 C,

0:05 + Plate Read

END

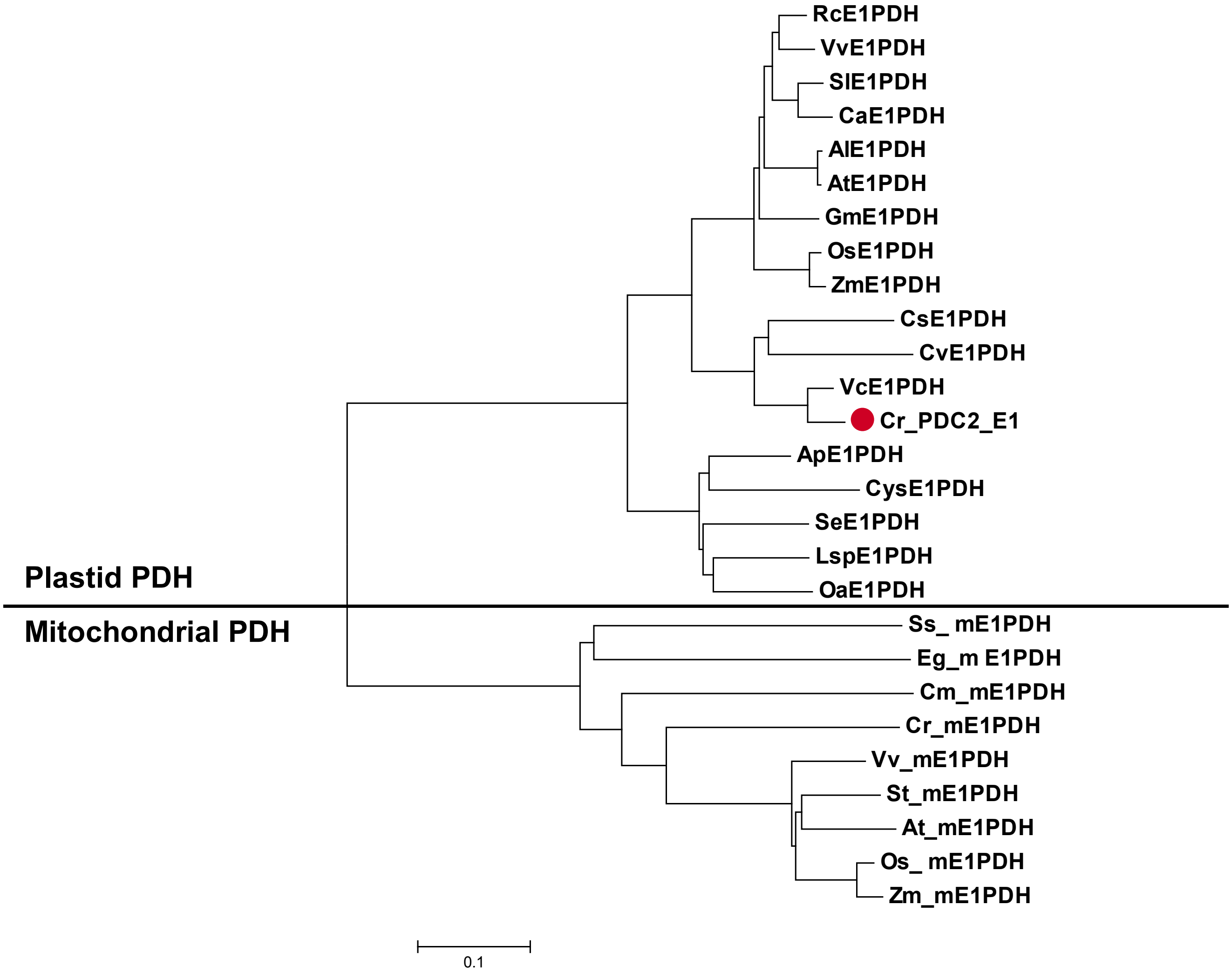

Figure S1. Phylogeny reconstruction of plastid and mitochondrial isoforms of pyruvate dehydrogenase (PDH) E1α subunit from plant, algal and bacterial species. The *C. reinhardtii* plastid localized protein is labeled with a red dot. Protein sequences were aligned by ClustalW and used by MEGA5.1 software for subsequent phylogenetic analysis by neighbor-joining method. GenBank accession numbers of the protein sequences used for the analysis: CsE1PDH – *C. subellipsoidea* EIE19084.1, VcE1PDH – *V. carteri* f. *nagariensis* XP_002950542.1, CvE1PDH – *C. variabilis* EFN53560.1, RcE1PDH – *R. communis* XP_002515074.1, GmE1PDH – *G. max* XP_003520883.1, SlE1PDH – *S. lycopersicum* XP_004251713.1, VvE1PDH – *V. vinifera* XP_002267676.1, CaE1PDH – *C. annuum* ACF17669.1, AlE1PDH – *A. lyrata* subsp.

lyrata XP_002889385.1, AtE1PDH – *A. thaliana* AT1G01090.1, OsE1PDH – *O. sativa* NP_001052065.1, ZmE1PDH – *Z. mays* NP_001140759.1, ApE1PDH – *A. platensis* NIES-39 BAI92470.1, LspE1PDH – *Lyngbya* sp. PCC 8106 ZP_01623820.1, OaE1PDH – *O. acuminata* PCC 6304 YP_007084537.1, CysE1PDH – *C. stanieri* PCC 7202 YP_007163963.1, SeE1PDH – *S. elongatus* PCC 7942 YP_172860.1, Cm_mE1PDH – *C. Midichloria mitochondrii IricVA* YP_004679065.1, St_mE1PDH – *S. tuberosum* P52903.1, Ss_ mE1PDH – *S. salar* P52903.1, Os_ mE1PDH – *O. sativa* NP_001048068.1, Vv_mE1PDH – *V. vinifera* XP_002274285.1, Zm_mE1PDH – *Z. mays* AAC72195.1, At_mE1PDH – *A. thaliana* AAA86507.1, Cm_mE1PDH – *C. merolae* strain 10D BAM83232.1, Eg_m E1PDH – *E. gracilis* emb|CAF05587.1, Cr-mE1PDH – *C. reinhardtii* Cre07.g337650.t1.2 (Phytozome).

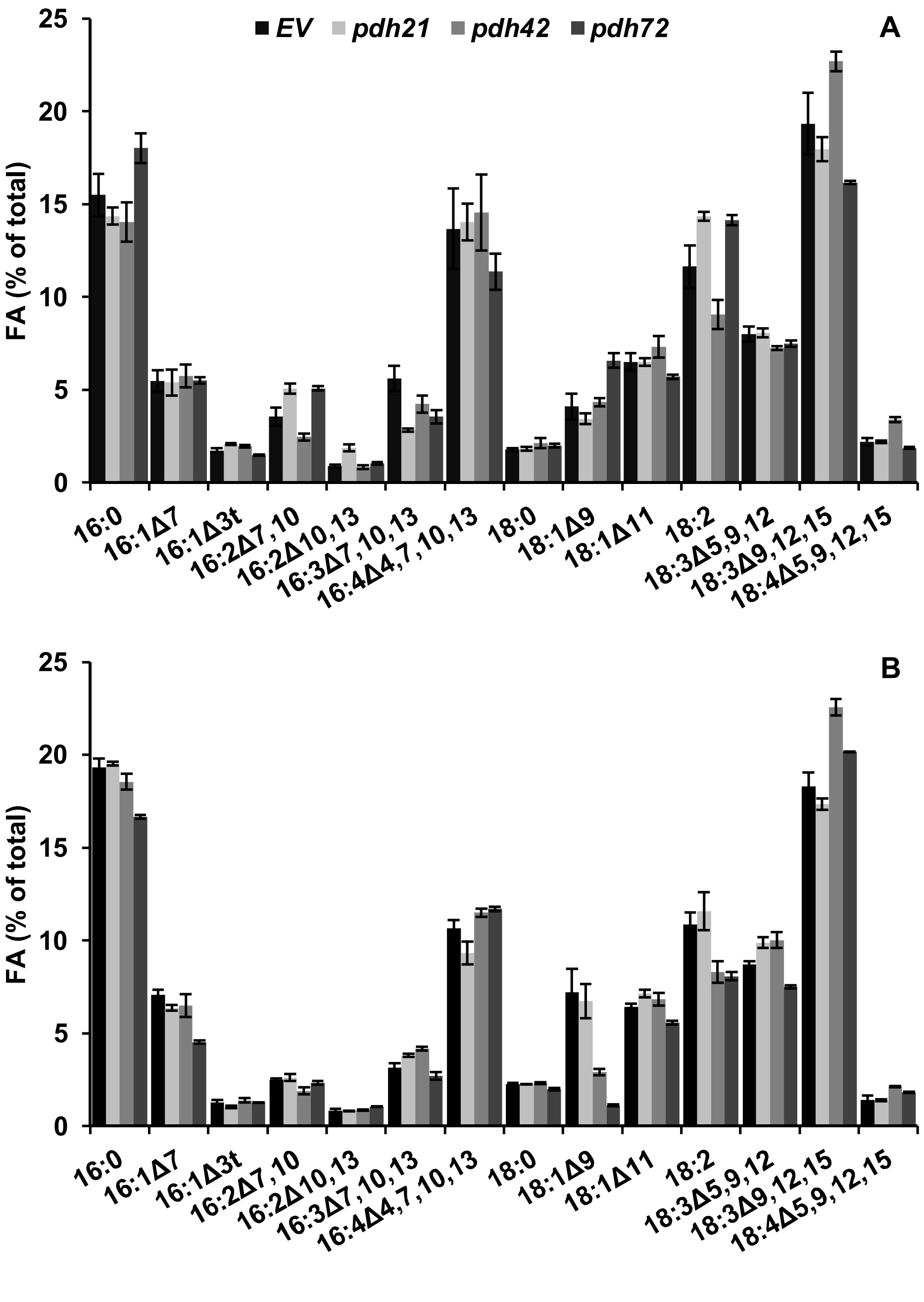

Figure S2. Effect of *PDC2_E1α* silencing on fatty acid composition of *Chlamydomonas* after six days cultivation. **(A)** In HSM medium. **(B)** In TAP medium. Values shown are means ± SD of two biological and two technical replicates (n = 4). EV values are means of two independent lines (n = 8).

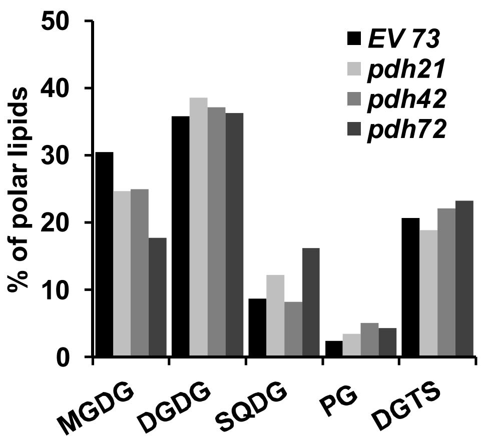

Figure S3. Effect of *PDC2_E1α* knockdown on distribution of acyl groups in major polar lipid classes (expressed as percentage of acyl groups) analyzed by gas chromatography. Pooled samples of three biological replicates were analyzed for each strain with no technical replicates. Phosphatidylinositol and phosphatidylethanolamine constituted less than 1% of total polar lipids. MGDG, monogalactosyldiacylglycerol; DGDG, digalactosyldiacylglycerol; SQDG, sulfoquinovosyl diacylglycerol; PG, phosphatidylglycerol; DGTS, 1,2-dipalmitoyl-*sn*-glycero-3-O-4'-[N,N,N-trimethyl(d9)]-homoserine. *Given the low content of phosphatidylethanolamine in the samples collected under nitrogen deprivation, an accurate analysis could not be performed.

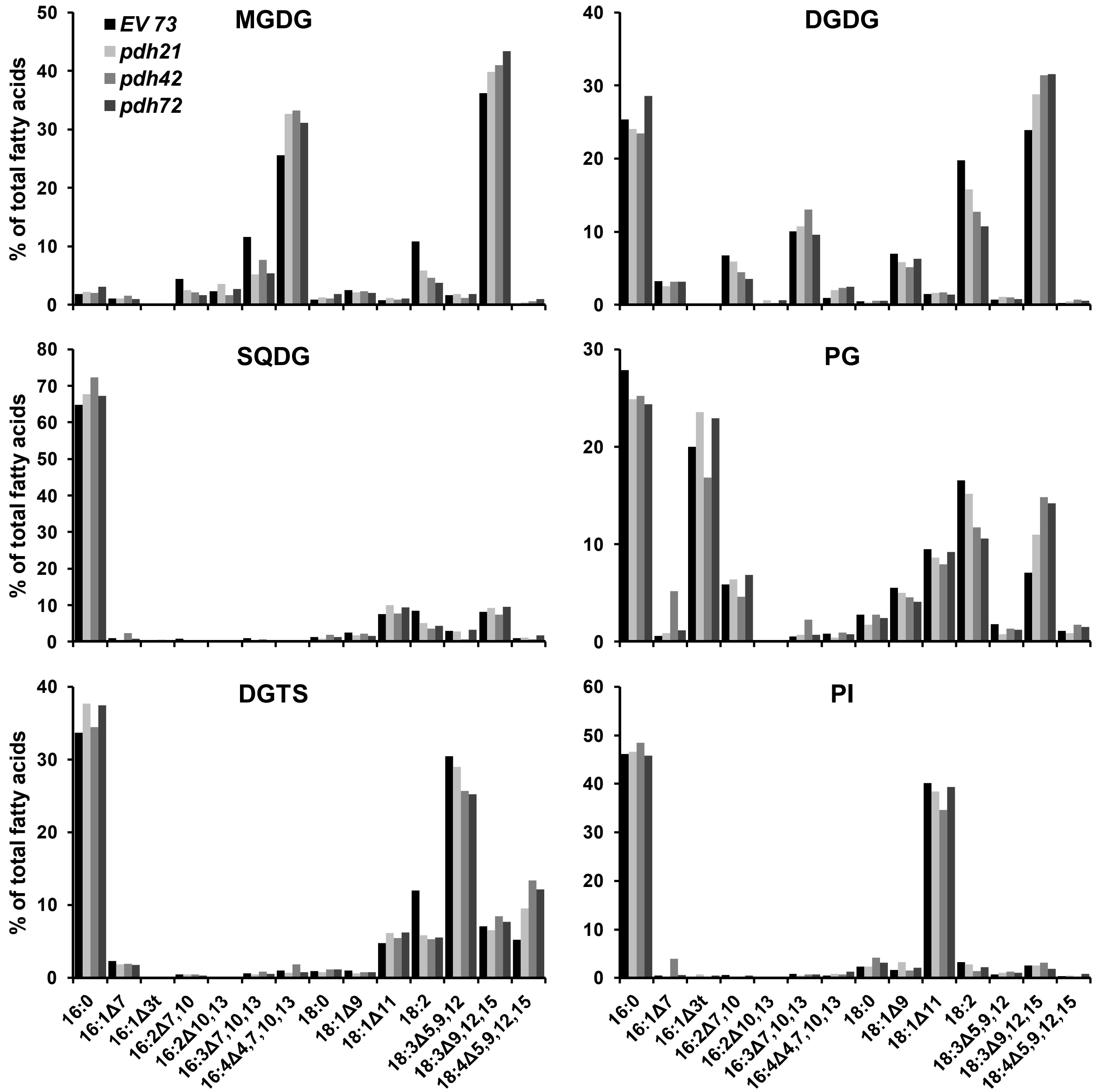

Figure S4. Distribution of fatty acids in individual lipids classes in EV and *pdh* lines. Total lipid extract was separated into neutral and polar lipids by TLC. Polar lipids were separated into individual classes by 2D TLC followed by GC analysis. Pooled samples of three biological replicates were analyzed for each strain with no technical replicates. The representative transformant lines are shown, similar results were obtained for other *pdh* mutants. MGDG, monogalactosyldiacylglycerol; DGDG,

digalactosyldiacylglycerol; SQDG, sulfoquinovosyl diacylglycerol; PG, phosphatidylglycerol; DGTS, 1,2-dipalmitoyl-*sn*-glycero-3-O-4'-[N,N,N-trimethyl(d9)]-homoserine; PI, phosphatidylinositol.

Table S3. Characteristic points of OJIP and derivation of the selected JIP-test parameters used in this work.

$F_{\mathrm{O}},\ F_{\mathrm{J}},\ F_{300}, F_{\mathrm{M}}$	Fluorescence yield at the points O, J, at 300 μs, and at the point of the maximum of OJIP (M)
$\Phi_{\mathrm{P}_0} = \frac{F_{\mathrm{M}} - F_{\mathrm{O}}}{F_{\mathrm{M}}} = \frac{F_{\mathrm{V}}}{F_{\mathrm{M}}}$	Maximum quantum yield of primary photochemistry (at t = 0)
$V_{\mathrm{J}} = \frac{F_{\mathrm{J}} - F_{\mathrm{O}}}{F_{\mathrm{M}} - F_{\mathrm{O}}}$	Normalized fluorescence at the point J
$\psi_0 = 1 - V_{\mathrm{J}}$	The probability of electron transfer from PS II to PQ pool
$M_0 = 4\frac{F_{300} - F_{\mathrm{O}}}{F_{\mathrm{M}} - F_{\mathrm{O}}}$	An approximation of the initial slope of OJIP attributed to Q_A reduction
$\Phi_{\mathrm{D}_0} = \frac{F_{\mathrm{O}}}{F_{\mathrm{M}}}$	Quantum yield of energy dissipation (at t = 0)
$\Phi_{\mathrm{E}_0} = \psi_0\left(1 - \frac{F_{\mathrm{O}}}{F_{\mathrm{M}}}\right)$	Quantum yield of electron transport (at t = 0)

Table S4. Characteristic parameters of chlorophyll fluorescence in the cells of *pdh* and EV lines of *C. reinhardtii*

**Parameter**	***pdh*21**			***pdh*42**			***pdh*72**			**EV60**			**EV73**		
	**TAP**	**HS**	**HS–N**	**TAP**	**HS**	**HS–N**	**TAP**	**HS**	**HS–N**	**TAP**	**HS**	**HS–N**	**TAP**	**HS**	**HS–N**
**Fo**	25*	88	45	30	82	64	25	82	75	31	105	66	31	90	64
**Fm**	90	304	128	109	282	173	91	229	160	112	409	234	127	363	221
ϕ_{P_0}	0.72	0.71	0.65	0.73	0.71	0.66	0.72	0.64	0.57	0.72	0.74	0.74	0.75	0.75	0.73
ϕ_{D_0}	0.25	0.29	0.35	0.25	0.29	0.34	0.24	0.36	0.43	0.28	0.27	0.26	0.22	0.25	0.27
ϕ_{E_0}	0.44	0.35	0.23	0.42	0.33	0.24	0.43	0.26	0.17	0.38	0.36	0.32	0.44	0.37	0.39

* Average values from two independent experiments performed in triplicate are presented; SD < 5% of the average

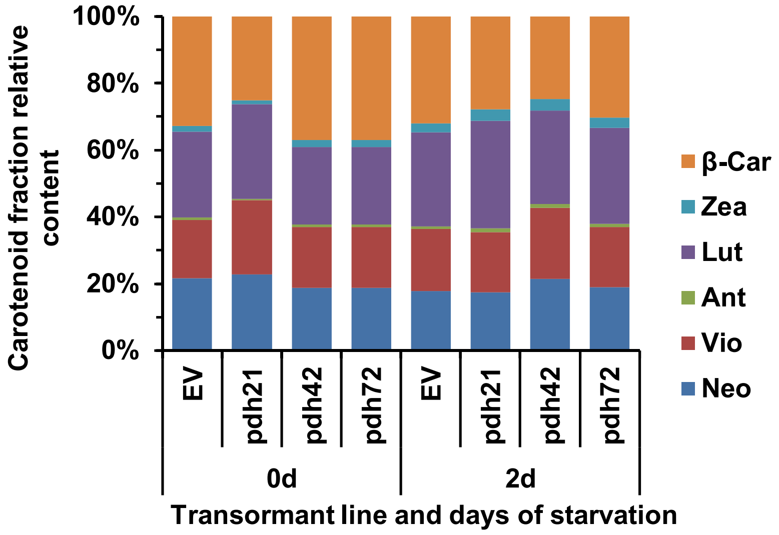

Figure. S5. Carotenoid composition of the transformed *C. reinhardtii* cells at the onset (0d; grown in TAP) and after two days of nitrogen starvation in HSM medium (2d). Ant—antheraxanthin, β-Car—β-carotene, Lut—lutein, Neo—neoxanthin + loroxanthin, Vio—violaxanthin. Data for HSM+N medium are not shown since they did not differ significantly from those for TAP (0d).

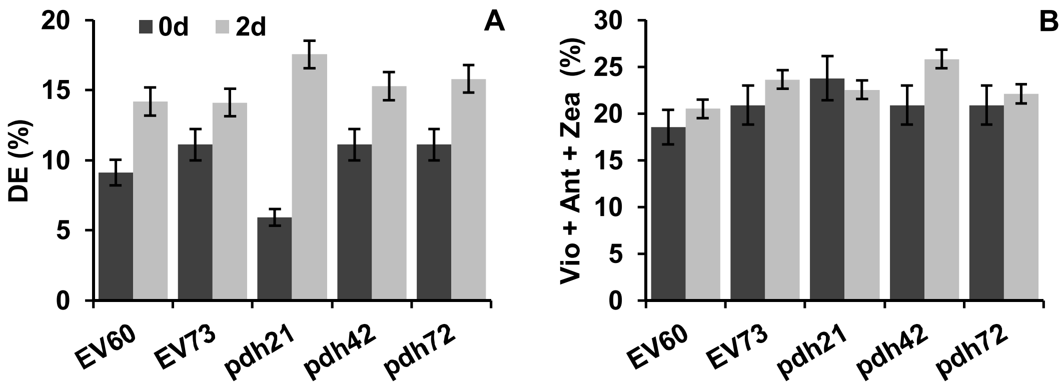

Figure S6. De-epoxidation state (A) of the violaxanthin cycle and violaxanthin cycle pigment pool size (B) of the transformed *C. reinhardtii* cells grown at the onset (0d, grown in TAP) and after two days of nitrogen starvation (2d) under photoautotrophic conditions in HSM.

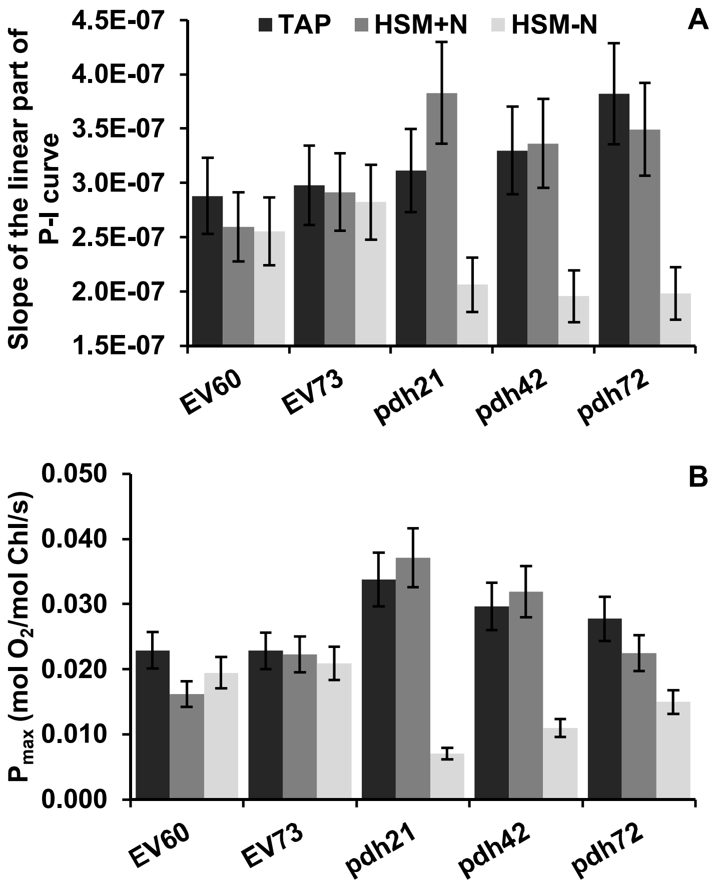

Figure S7. Photosynthetic activity of the transformed *C. reinhardtii* cells grown in TAP, HS+N or HS-N media as manifested by P-I curves recorded via oxygen evolution. (A) slope of the linear part of the P-I curve; (B) maximum rate of photosynthesis.

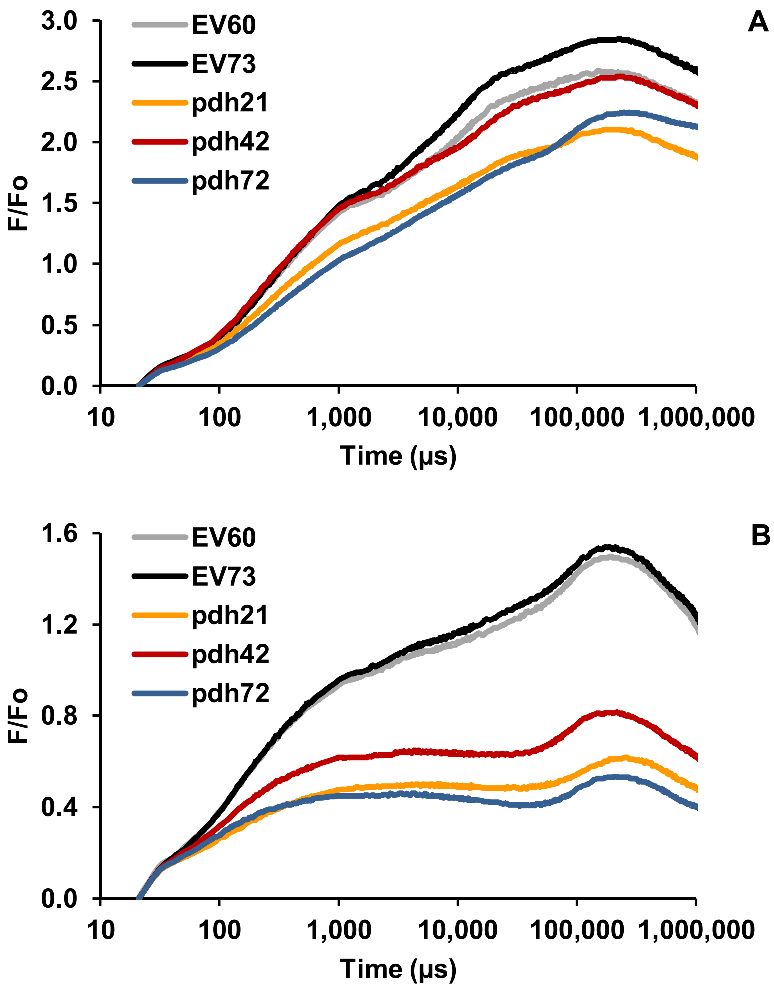

Figure S8. Typical curves of chlorophyll fluorescence induction (OJIP) recorded in the initial culture grown in TAP (A) or after two days of nitrogen starvation in HSM medium (B) of the transformed *C. reinhardtii* cells with a standard FP100s. OJIP were induced by irradiation (3000 μE · m^{-2} · s^{-1}) of the samples adapted in the dark for 10 min. Data for HS+N medium are not shown since they did not differ significantly from those for the initial culture.
